# Association Between the Methicillin Resistance of *Staphylococcus aureus* Isolated from Slaughter Poultry, Their Toxin Gene Profiles and Prophage Patterns

**DOI:** 10.1007/s00284-018-1518-9

**Published:** 2018-05-29

**Authors:** Agnieszka Marek, Ewelina Pyzik, Dagmara Stępień-Pyśniak, Renata Urban-Chmiel, Łukasz S. Jarosz

**Affiliations:** 1 Sub-Department of Preventive Veterinary and Avian Diseases, Institute of Biological Bases of Animal Diseases, Faculty of Veterinary Medicine, University of Life Sciences in Lublin, Akademicka 13, 20-950 Lublin, Poland; 2 Department of Epizootiology and Clinic of Infectious Diseases, Faculty of Veterinary Medicine, University of Life Sciences in Lublin, Głęboka 30, 20-612 Lublin, Poland

## Abstract

In this work, 85 strains of *Staphylococcus aureus* were isolated from samples taken from slaughter poultry in Poland. Attempts were made to determine the prophage profile of the strains and to investigate the presence in their genome of genes responsible for the production of five classical enterotoxins (A–E), toxic shock syndrome toxin (TSST-1), exfoliative toxins (ETA and ETB) and staphylokinase (SAK). For this purpose, multiplex PCR was performed using primer-specific pairs for targeted genes. The presence of the *mec*A gene was found in 26 strains (30.6%). The genomes of one of the methicillin-resistant *S. aureus* (MRSA) strains and two methicillin-sensitive *S. aureus* (MSSA) strains contained the gene responsible for the production of enterotoxin A. Only one MRSA strain and two MSSA strains showed the presence of the toxic shock syndrome toxin (*tst*) gene. Only one of the MSSA strains had the gene (*eta)* responsible for the production of exfoliative toxins A. The presence of the staphylokinase gene (*sak*) was confirmed in 13 MRSA strains and in 5 MSSA strains. The study results indicated a high prevalence of prophages among the test isolates of *Staphylococcus aureus*. In all, 15 prophage patterns were observed among the isolates. The presence of 77-like prophages incorporated into bacterial genome was especially often demonstrated. Various authors emphasize the special role of these prophages in the spread of virulence factors (staphylokinase, enterotoxin A) not only within strains of the same species but also between species and even types of bacteria.

**Electronic supplementary material:**

The online version of this article (10.1007/s00284-018-1518-9) contains supplementary material, which is available to authorized users.

## Introduction

*Staphylococcus aureus* has the ability to induce severe diseases of animals and humans, that are associated with numerous virulence factors produced by these bacteria, such as toxins, cell adhesins and secreted exoproteins (coagulase, toxic shock syndrome toxin-1, staphylokinase and others) [1–5]. Staphylococcal enterotoxins (SEs) and toxic shock syndrome toxin-1 (TSST-1) are exotoxins that belong to the superantigen family [6, 7]. The mechanism of action of superantigens involves the ability to induce non-specific stimulation of T lymphocyte proliferation and cytokine secretion. The accumulation of cytokines in the mammalian body can lead to toxic shock. Another consequence of the presence of enterotoxins in the body is their enterotoxicity, resulting in food poisoning. A single bacterial strain may produce any of these toxins separately or in various combinations [2, 8].

Staphylokinase (SAK) a protein produced by certain *S. aureus* strains is a fibrin-specific activator of human plasminogen. During the infection process, staphylokinase disrupts phagocytosis of bacterial cells, and fibrinolysin, a component of staphylokinase, facilitates the penetration of staphylococci into tissues and their proteolytic degradation [9]. The ability of *S. aureus* strains to produce staphylokinase can be exploited to trace the ecological origin of a strain. It has been observed that *S. aureus* strains of human biotype can produce enterotoxins much more frequently than strains of animal biotypes. However, some researchers claim that strains derived from poultry are also capable of producing this protein [3].

Mobile genetic elements (MGEs), such as bacteriophages, plasmids, pathogenicity islands, transposons or insertion sequences, encode putative virulence factors and molecules that confer the ability to produce enterotoxins, TSST-1, exfoliative toxins or staphylokinase [10–13]. The vast majority of bacteria contain prophages integrated into their chromosome or as extra-chromosomal elements. Detection of staphylococcal prophages by multiplex PCR is comparable in sensitivity to detection by hybridization of restriction fragments [14]. A key role in the pathogenesis and virulence of the *S. aureus* is attributed to temperate bacteriophages of the *Siphoviridae* family belonging to the order *Caudovirales*. Based on their lytic activity, morphology and serological properties, phages of the *Siphoviridae* family are classified into six phage types: 3A-like virus, 11-like virus, 77-like virus and 187-like virus. Phages of type 77-like fall into two serological subgroups, Fa and Fb, which can be present at the same time as prophages in the genome of bacteria [15]. Phages of the Twort-like type are related to lytic phages and belong to the family *Myoviridae*. Bacteriophages, via lysogenic conversion and participation in the spread of pathogenic islands, are believed to contribute to *S. aureus* variability and the formation of highly virulent strains [16]. They can also influence the adaptation of *S. aureus* to its hosts, both human and domesticated animals, by providing new genetic information or facilitating the loss of unnecessary DNA in the new ecological niche. It has been demonstrated that *S. aureus* strains of animal origin may be susceptible to the same bacteriophages as human strains, and thus via phage they can acquire the virulence factors characteristic for human strains [11].

Despite efforts to reduce the use of antibiotics in poultry production in the past few years, there has been a steady increase in the incidence of bacterial infections with multiple-resistant strains of *Staphylococcus* species in poultry flocks. There has also been an enormous increase in the number of methicillin-resistant (MRSA) strains [17, 18]. Resistance to beta-lactam antibiotics is usually dependent on the presence of the *mec*A gene, which encodes the low-affinity penicillin-binding protein (PBP)-designated PBP2a and makes the staphylococci resistant to almost all antibiotics of this group (penicillins, almost all cephalosporins and carbapenems), of which many are still widely used in both human and veterinary medicine [19, 20]. The presence of virulence factors and antibiotic resistance genes in isolates of *S. aureus* indicates the risk carried by a particular strain.

The objective of this study was to determine the prevalence of methicillin-resistant (MRSA) and methicillin-sensitive *S. aureus* (MSSA) in samples taken from chicken and turkey broilers in Poland, and the associations between the methicillin resistance of *S. aureus* strains isolated from poultry, their toxin gene profiles and their ability to produce staphylokinase. Because the vast majority of *S. aureus* contain prophages integrated into their chromosome, their prophage patterns were identified as well. Gene polymorphism of twenty-six MRSA isolates by Pulsed-field gel electrophoresis (PFGE) with restriction enzymes *Sma*I and *Apa*I was performed as well.

## Methods

### Sample Collection

The study was conducted on material derived from broiler chickens and turkeys farms located in the area of central-western Poland between December 2013 and November 2015. During this time, samples from 153 flocks were collected. Randomly selected birds showing clinical signs of disease from each flock were examined. Three to five specimens from the affected organs were taken from each bird. A total of 569 samples from broilers and 446 samples from turkeys were collected. The samples were taken from internal organs (heart, liver, tarsal joints and bone marrow) of birds aged 1 day to 6 weeks (chickens) or 20 weeks (turkeys). The samples were taken from birds showing the following clinical symptoms: increased mortality, dermatitis and cellulitis, lameness and arthritis, decreased weight gain and omphalitis and yolk sac infections. The size of the flocks from which the samples were collected ranged from 8000 to 44000 birds.

The material collected (samples of internal organs) was plated on a blood agar medium (Blood LAB-AGAR, Biocorp, Poland) and Chapman selective medium (Mannitol Salt LAB-AGAR, Biocorp, Poland) and incubated under aerobic conditions at 37 °C for 24–48 h, depending on the rate of growth of the bacteria. Single colonies were transferred to blood agar in order to isolate pure bacterial cultures and a preliminary bacteriological characterization was made of the isolated flora, involving Gram’s staining, cell morphology and motility using microscope and type of haemolysis. In this study, quantitative measurement of the colony was not performed.

Isolated bacteria were stored for further testing at − 85 °C in 50% (v/v) glycerol in brain heart infusion broth (BHIB; Sigma).

### Statement of Human and Animal Rights

All procedures performed in studies involving human participants were in accordance with the ethical standards of the institutional and/or national research committee and with the 1964 Helsinki Declaration and its later amendments or comparable ethical standards.

### Characterization of Bacterial Strains

The identification of all *Staphylococcus* strains was carried out using mass spectrometry MALDI-TOF MS using the IVD MALDI Biotyper (Bruker Daltonik, Bremen, Germany) as described by Marek et al. [21].

The susceptibility of 11 antibiotics was tested using standard disc diffusion method on Mueller–Hinton agar plates (CM0337B, Oxoid, UK) using a bacterial suspension with the turbidity adjusted to a 0.5 McFarland standard. The susceptibility of bacteria was determined for the following agents (Oxoid, England): amoxicillin 25 µg (AML25); amoxicillin + clavulanic acid 20 + 10 µg (AMC30); ampicillin 10 µg (AMP10); penicillin G 10 units (P10); cefoxitin 30 µg (FOX30); clindamycin 2 µg (DA2); chloramphenicol 30 µg (C30); erythromycin 15 µg (E15); gentamicin 10 µg (CN10); tetracycline 30 µg (TE30); trimethoprim–sulphamethoxazole 1:19, 25 µg (SXT25). The categories susceptible, intermediate resistant or resistant were assigned on the basis of the Guidelines for Susceptibility Testing [20]. The Minimum Inhibitory Concentrations (MIC) for oxacillin were additionally evaluated by the broth microdilution method [20]. For oxacillin, *S. aureus* strains showing MIC of ≥ 4 µg/ml were determined to be MRSA. For quality control, *S. aureus* ATCC 25923, *Escherichia coli* ATCC 25922 and *E. faecalis* ATCC 29212 were used in the microdilution tests.

### Bacterial DNA Extraction

Total DNA was extracted from the collected strains inoculated individually on blood agar and incubated at 37 °C/24 h. The Novabeads Bacterial DNA kit (Novazym Poland) was used for DNA extraction according to the manufacturer’s protocol.

### Detection of the *mec*A Gene

The identification of all MRSA isolates was confirmed by multiplex PCR (set A) targeting the *mec*A gene encoding methicillin resistance. To verify the efficiency of the amplification, an internal control primer pair targeting an *S. aureus*-specific *nuc* region was amplified. PCR for *mec*A and *nuc* gene was carried out by the methodology described by Brakstad et al. and Murakami et al. [18, 22]. Isolates of *S. aureus* that were *mec*A and *nuc* positive were considered as MRSA.

### Prevalence of Toxins and *sak* Genes in *S. aureus* and MRSA Isolates

A multiplex PCR assay for detection of genes for staphylococcal enterotoxins A to E (*sea, seb, sec, sed* and *see*)—set B, and for toxic shock syndrome toxin 1 (*tst*) and exfoliative toxins A and B (*eta* and *etb*)—set C, was developed using eight pairs of primers (Table 1). The conditions of the multiplex PCR reaction were taken from the study by Mehrota et al. [23].The PCR primers used to amplify the *sak* gene are listed in Table 1. The conditions of the PCR reaction were taken from the study by Sung et al. [24].

**Table 1:** Nucleotide sequences and sizes of PCR products of methicillin resistance, thermonuclease, enterotoxins (A-E), TSST-1, exfoliative toxins and staphylokinase

Primer*	Oligonucleotide sequence (5′–3′)**	Gene	Size of amplified product (bp)	Control strain	References
NUC-1	GCGATTGATGGTGATACGGTT	*nuc*	270	ATCC43300	[22]
NUC-	AGCCAAGCCTTGACGAACTAAAGC				
MEC-1	AAAATCGATGGTAAAGGTTGGC	*mecA*	533		[18]
MEC-2	AGTTCTGGCACTACCGGATTTGC				
ESA1	ACGATCAATTTTTACAGC	*sea*	544	FRI913	[10]
ESA2	TGCATGTTTTCAGAGTTAATC				
ESB1	GAATGATATTAATTCGCATC	*seb*	416	ATCC13566	[25]
ESB2	TCTTTGTCGTAAGATAAACTTC				
ESC1	GACATAAAAGCTAGGAATTT	*sec*	257	FRI913	[10]
ESC2	AAATCGGATTAACATTATCCA				
ESD1	TTACTAGTTTGGTAATATCTCCTT	*sed*	334	FRI151m	[26]
ESD2	CCACCATAACAATTAATGC				
ESE1	ATAGATAAAGTTAAAACAAGCAA	*see*	170	FRI913	[27]
ESE2	TAACTTACCGTGGACCC				
GTSSTR-1	ACCCCTGTTCCCTTATCATC	*tst*	326	FRI1169	[28]
GTSSTR-2	TTTTCAGTATTTGTAACGCC				
GETAR-1	GCAGGTGTTGATTTAGCATT	*eta*	93	CCM7056	[29]
GETAR-2	AGATGTCCCTATTTTTGCTG				
GETBR-1	ACAAGCAAAAGAATACAGCG	*etb*	226	CCM7056	[29]
GETBR-2	GTTTTTGGCTGCTTCTCTTG				
SAK-1	TGAGGTAAGTGCATCAAGTTCA	*sak*	403	ATCC25923	[30]
SAK-2	CCTTTGTAATTAAGTTGAATCCAGG				

*The sets of primers were synthesized by Genomed S.A, Poland

**The concentration of primers was 0.04 µmol

### Multiplex PCR for Detection of Prophages in *S. aureus* Strains

A multiplex PCR assay (set D) for detection of DNA sequences specific for 3A-like, 11-like, 77-like, 187-like and Twort-like phages was developed using eight pairs of primers (Table 2). The conditions of the multiplex PCR reaction were taken from the study by Pantůček et al. [31].

**Table 2:** Multiplex PCR^*^. Primer sequence of staphylococcal phage type, PCR product length and type of protein

Phage type	Primer***	Primer sequence (5′–3′)**	PCR product length (bp)	Sequence coding for
3A-like phage	SGA1	TATCAGGCGAGAATTAAGGG	744	Tail fibres
	SGA2	CTTTGACATGACATCCGCTTGAC		
11-like phage	SGB1	ACTTATCCAGGTGGYGTTATTG	405	Hypothetical tail protein
	SGB2	TGTATTTAATTTCGCCGTTAGTG		
77-like phage	SGF1	CGATGGACGGCTACACAGA	155	Hypothetical tail protein
	SGF2	TTGTTCAGAAACTTCCCAACCTG		
	SGFa1	TACGGGAAAATATTCGGAAG	548	Packaging protein
	SGFa2	ATAATCCGCACCTCATTCCT		
	SGFb1	AGACACATTAAGTCGCACGATAG	147	Packaging protein
	SGFb2	TCTTCTCTGGCACGGTCTCTT		
187-like phage	SGL1	GCTTAAAACAGTAACGGTGACAGTG	648	Hypothetical capsid protein
	SGL2	TGCTACATCATCAAGAACACCTGG		
Twort-like phage	SGD1	TGGGCTTCATTCTACGGTGA	331	Major capsid protein
	SGD2	GTAATTTAATGAATCCACGAGAT		

*S. aureus strain NCTC 8325 was used as positive control

**Nucleotide sequences were derived from the published sequences by Pantůček et al [31]

***The concentration of primers was 0.04 µmol

### Pulsed-field Gel Electrophoresis (PFGE)

The genetic relatedness of MRSA isolates was investigated by PFGE of total DNA digested with *Sma*I or *Apa*I restriction endonucleases [32]. *S. aureus* non-typeable with *Sma*I were subjected to *Apa*I-PFGE and run for 20 h at 6V/cm using pulsed time ranging from 2 to 5 s. The *Sma*I or *Apa*I fragments were electrophoretically separated in a 1% (w/v) agarose (Sigma-Aldrich, Poland) gel using the CHEF Mapper System (BIO-RAD, Poland). The macrorestriction patterns were examined by cluster analysis using NTSYSpc ver. 2.02 software (Exeter Software Ltd, USA). The similarity distances between pulsotypes (PFGE patterns) were calculated using the Dice coefficient, and the dendrogram was based on the unweighted pair group method with arithmetic average (UPGMA). According to the criteria proposed by Tenover et al., isolates whose PFGE pattern differed in more than six restriction fragments (bands) were genetically unrelated and were assigned to different pulsotypes, named as Arabic letters. Isolates were considered to be related if their pulsotype differed in no more than six restriction bands (subtype) and were indicated with the major lettering type followed by a number [33]. *Sma*I- and *Apa*I-generated pulsotypes were distinguished by capital- and lower-case Arabic letters, respectively.

## Results

### Sample Collection

A total of 567 bacterial strains belonging to the genus *Staphylococcus* were isolated from the material tested. The *Staphylococcus* strains isolated from the samples belonged to 24 species. Among 24 *Staphylococcus* species, 85 strains of *S. aureus* were identified. The remaining strains belonged to the species *S. cohnii* (27.7%), *S. lentus* (17%), *S. chromogenes* (8.4%), *S. equorum* (6.5%), *S. saprophyticus* (4.4%), *S. sciuri* (3.9%), *S. hominis* (3.2%), *S. xylosus* (3.2%), *S. arlettae* (2.4%), *S. simulans* (1%), *S. felis* (0.9%), *S. vitulinus* (0.9%), *S. delphini* (0.7%), *S. epidermidis* (0.7%), *S. haemolyticus* (0.7%), *S. condimenti* (0.5%), *S. warneri* (0.5%), *S. alactolyticus* (0.4%), *S. carnosus* (0.4%), *S. capitis* (0.4%), *S. hyicus* (0.4%), *S. lugdunensis* (0.4%), *S. schleiferi* subsp. *coagulans* (0.4%).

PCR confirmed the presence of the *nuc* gene in all 85 (100%) *S. aureus* strains.

### Prevalence of MRSA Strains

PCR confirmed the presence of a 533 bp product characteristic of the presence of the *mec*A gene in 26 (30.6%) strains.

### Phenotypic Susceptibility of the Isolated Bacteria to Selected Antimicrobial Agents

The minimum inhibitory concentration (MIC) against oxacillin showed that 24 isolates of *S. aureus* (28.2%) were resistant to this antibiotic. All strains showing a MIC value indicating resistance to oxacillin also possessed the *mec*A gene.

As a result of the susceptibility testing of the isolated *S. aureus* strains to 11 selected antimicrobial agents, 100% susceptibility in *in vitro* conditions for cefoxitin was observed among of 59 MSSA strains. A relatively high percentage of MSSA strains were found to be susceptible to clindamycin (96.6%), trimethoprim–sulphamethoxazole (96.6%), chloramphenicol (93.3%), amoxicillin + clavulanic acid (93.2%) and amoxicillin (89.8%). Considerably more MSSA isolates exhibited resistance to gentamicin (18.6%) and tetracycline (28.8%). Over half of the MSSA strains were resistant to the other three antimicrobial agents, with the highest percentage of strains, 68.3% resistant to penicillin G. Among MRSA isolates, 100% *in vitro* susceptibility was not observed in any of the eleven antimicrobial agent applied. All MRSA strains were resistant to penicillin G and ampicillin. A high percentage of strains were resistant to amoxicillin (96.2%), cefoxitin (85%), amoxicillin + clavulanic acid (84.7%), tetracycline (84.7%), erythromycin (80.8%) and clindamycin (73%). Detailed data are presented in Table 3.

**Table 3:** Phenotypic^*^, antimicrobial resistance of *S. aureus* strains isolated from broiler chickens and turkeys

Antibiotic	MRSA strains *n* = 26			MSSA strains *n* = 59		
	R	I	Resistance rate (%)	R	I	Resistance rate (%)
Amoxicillin	23	2	96.2	5	1	10.2
Amoxicillin + clavulanic acid	12	10	84.7	4	–	6.8
Ampicillin	24	2	100	32	–	54.2
Penicillin G	26	–	100	38	2	68.3
Cefoxitin	22	–	85	–	–	–
Clindamycin	14	5	73	–	2	3.4
Chloramphenicol	6	1	26.9	1	3	6.7
Erythromycin	17	4	80.8	31	–	52.5
Gentamicin	5	–	19.2	10	1	18.6
Tetracycline	20	2	84.7	11	6	28.8
Trimethoprim–sulphamethoxazole	3	1	15.3	2	–	3.4

The resistance rate was calculated as the number of intermediate and resistant isolates divided by the total number of isolates

*R* resistant, *I* intermediate

^*^The susceptibility o11 antibiotics was tested using standard disc diffusion method on Mueller–Hinton agar plates

### Prevalence of Toxin Genes in *S. aureus* and MRSA Isolates

The results of the multiplex PCR for five classical enterotoxins (A–E) showed that the genome of one of the MRSA strains contained the gene responsible for the production of enterotoxin A. Four strains carried the gene responsible for the production of enterotoxin B and one strain contained the gene responsible for the production of enterotoxin D. None of the MRSA strains had genes responsible for the production of enterotoxins C and E (Table 4).

**Table 4:** Prophage content and prevalence of staphylokinase and toxins genes of the 26 MRSA *S. aureu*s isolates estimated by multiplex PCR

Lysogenic type	Number of MRSA strains (*n* = 26)	(%)	Presence of the gene				PFGE pulsotype
			*sea* *seb* *sec* *sed* *see*	*tst*	*eta* *etb*	*sak*	
Single lysogenic strains							
11-like (SGB)	2	7.7	–	–	–	–	A, a2
77-like (SGFa)	4	15.4	–	–	–	4	a1, a4, a5, a8
77-like (SGFb)	4	15.4	*seb* (*n* = 1)			2	a1, a2
Double lysogenic strains							
3A (SGA)—11 (SGB)	1	3.8	–	–	–	–	d
3A(SGA)—77a (SGFa)	4	15.4	*sea* (*n* = 1) *seb* (*n* = 1)	1	–	2	a3, a11, a12, c
11 (SGB)—77b (SGFb)	2	7.7	–	–	–	1	a1, a9
77a (SGFa)—77b (SGFb)	3	11.5	*sed*(*n* = 1)			1	a1, a6, a7
77b (SGFb)—187 (SGL)	1	3.8	*seb*(*n* = 1)	–	–	1	B
Triple lysogenic strains							
3A (SGA)—11 (SGB)—77a (SGFa)	1	3.8	–	–	–	–	C
3A (SGA—77a(SGFa)- 77b (SGFb)	2	7.7	*seb* (*n* = 1)	–	–	1	a1, b
11 (SGB)–77a(SGFa)—77b (SGFb)	2	7.7	–	–	–	1	a10, a13

*MRSA* methicillin-resistant *S. aureus, sea, seb, sec, cec, see* enterotoxin A,B,C,D,E, *tst* toxic shock syndrome toxin-1, *eta, etb* exfoliative toxins A, B, *sak* staphylokinase

In the case of MSSA strains (*n* = 59), the presence of two strains capable of producing enterotoxin A was confirmed. Five strains showed the presence of the gene responsible for the production of enterotoxin B, two strains had genes responsible for the production of enterotoxin C and one strain had the gene responsible for the production of enterotoxin D. None of the MSSA strains carried the gene responsible for the production of enterotoxin E (Table 5; Fig. 1 in supplementary materials).

**Table 5:** Prophage content and prevalence of staphylokinase and toxins genes of the 59 MSSA *S. aureu*s isolates estimated by multiplex PCR

Lysogenic type	Number of MSSA strains (*n* = 59) with respective PCR pattern	(%)	Presence of the gene				
			sea seb sec sed see	tst	eta etb	sak	
Single lysogenic strains							
3A-like (SGA)	5	8.5	sea (*n* = 1)				
11-like (SGB)	11	18.6		1			
77-like (SGFa)	3	5.1					
77-like (SGFb)	7	11.9	seb (*n* = 1)				
Double lysogenic strains							
3A (SGA)—11 (SGB)	7	11.9	seb (*n* = 1) sec (*n* = 1)				1
3A(SGA)—77a (SGFa)	2	3.4	sea (*n* = 1)				1
11 (SGB)—77b (SGFb)	7	11.9	seb (*n* = 1)		A (*n* = 1)		
11 (SGB)—77a (SGFa)	2	3.4					
77a (SGFa)—77b (SGFb)	4	6.8	sed (*n* = 1)				
Triple lysogenic strains							
3A (SGA)- 11 (SGB)—77a (SGFa)	3	5				2	
3A (SGA)- 11 (SGB)—77b (SGFb)	4	6.8		1			
11 (SGB)—77a (SGFa)—77b (SGFb)	3	5	seb (*n* = 1)				
Quadruple lysogenic strains							
3A-like (SGA)—11-like (SGB)—77a (SGFa)—77b (SGFb)	1	1.7	seb (*n* = 1) sec (*n* = 1)			1	

*MSSA* methicillin-sensitive *S. aureus, sea, seb, sec, cec, see* enterotoxin A,B,C,D,E, *tst* toxic shock syndrome toxin-1, *eta, etb* exfoliative toxins A, B, *sak* staphylokinase

Regarding TSST-1 and exfoliative toxins A and B, only one MRSA strain and two MSSA strains showed the presence of the *tst* gene. None of the MRSA strains had genes responsible for the production of exfoliative toxins A and B, and only one of the MSSA strains had the *eta* gene (Tables 4, 5 and Fig. 3 in supplementary materials).

### Detection of the Staphylokinase (*sak*) Gene

The presence of the *sak* gene was demonstrated in 13 strains of MRSA and in 5 strains of MSSA. Detailed data are shown in Tables 4 and 5.

### Prophage Pattern in *S. aureus* Strains

Multiplex PCR successfully distinguished the prophage 3A-like (SGA), 11-like (SGB) and 77-like (SGF) serogroups, the 77-like a (SGFa) and b (SGFb) subgroups and 187-like (SGL).

In our study, four serotypes and two subtypes of prophages were detected. Furthermore, all MRSA and MSSA isolates contained at least one prophage incorporated in their genome. A total of 11 prophage patterns were identified among MRSA strains and 13 prophage patterns among MSSA strains. The most prevalent prophage patterns or lysogenic types among MRSA isolates were single lysogenic 77-like (Fa subtype), 77-like (Fb subtype) and double lysogenic 3A-like-77-like (Fa subtype). Among MSSA isolates, the most prevalent prophage pattern was 11-like, followed by single lysogenic 77-like (Fb subgroup), double lysogenic 3A-like-11-like and 11-like-77-like (Fb subgroup); detailed data are presented in Tables 4 and 5 and Fig. 2 in supplementary materials. Prophages of the Twort type were not identified in any of the 85 *S. aureus* strains tested, while prophage 187-like was identified in the genome of one of the MRSA strains.

### Pulsed-Field Gel Electrophoresis (PFGE)

**Fig. 1:**
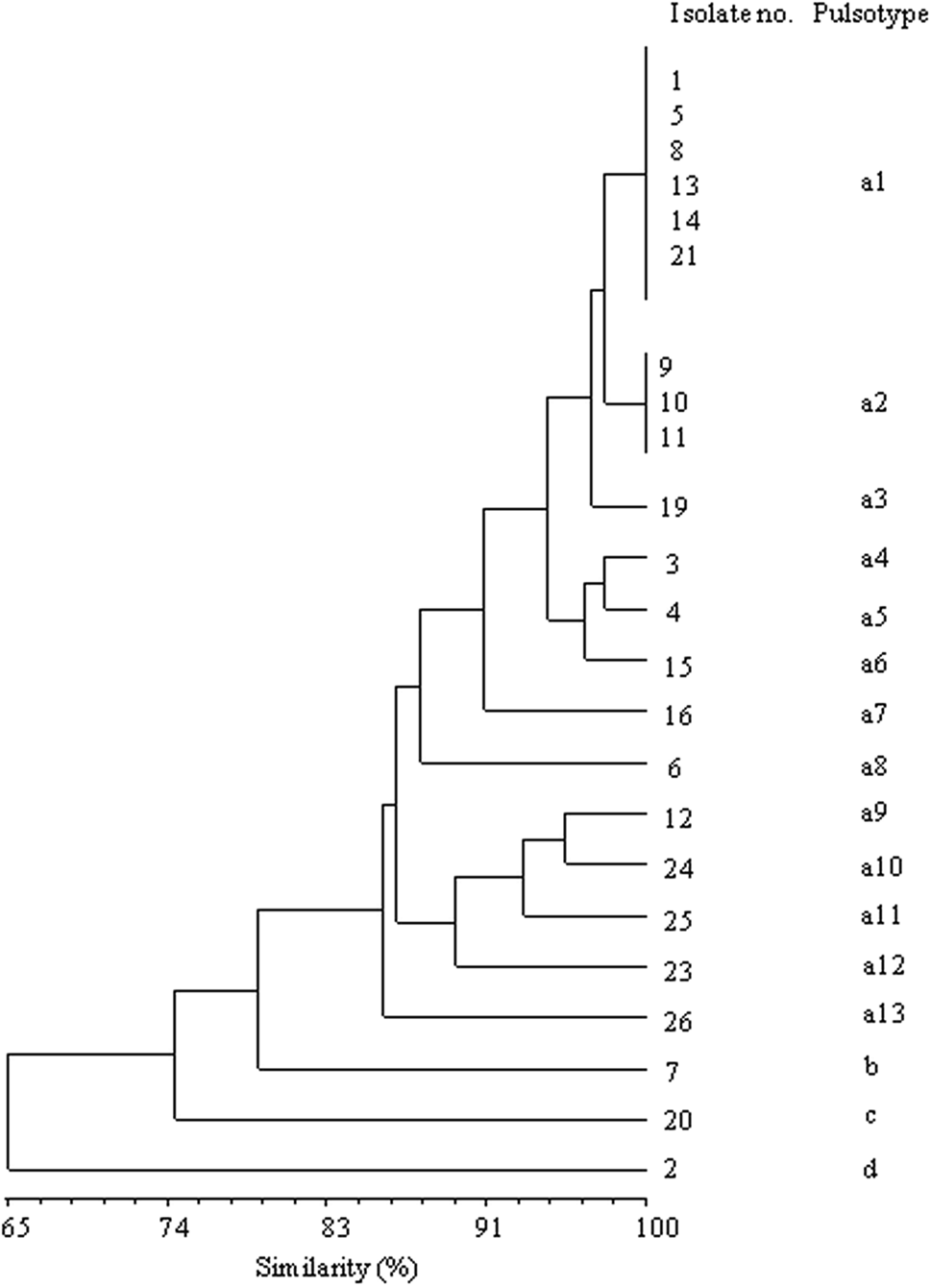
Dendrogram showing genetic similarity among twenty-three MRSA isolates digested with *Apa*I restriction endonuclease

Only three of the MRSA isolates were digested by *Sma*I enzyme and belonged to three different pulsotypes named as A, B and C (Table 4 and Figs. 4, 5 in supplementary materials). However, all twenty-six MRSA isolates were typeable using the restriction enzyme *Apa*I. The macrorestriction profiles obtained, following enzyme *Apa*I digestion, are presented in Figs. 6, 7 and 8 in supplementary materials. Analysis of the phylogenetic relationship between twenty-three of MRSA strains distinguished 16 macrorestriction profiles following digestion with *Apa*I endonuclease. Six isolates belonging to the pulsotype a1 and three isolates belonging to the pulsotype a2 showed the same *Apa*I-PFGE patterns (Fig. 1). The genetic similarity of the remaining 14 MRSA strains ranged from 65 to 98% (Fig. 1).

## Discussion

The threat of *Staphylococcus aureus* is due to its ease of transmission between animals and humans and its pathogenicity. It has been demonstrated that livestock-associated *S. aureus* strains (LA-MRSA) originated in humans and vice versa [34, 35]. Here, we characterized the presence of a group of genes responsible for toxin production and methicillin resistance in *S. aureus* isolated from slaughter poultry. Determination of the oxacillin MIC is a method recommended by the CLSI for the detection of meticillin resistance in routine testing; however, some recent studies have reported low sensitivity and low specificity of oxacillin compared with cefoxitin [20]. Therefore, cefoxitin is considered to be a better predictor than oxacillin for the detection of heteroresistance because it is a stronger inducer of penicillin-binding protein 2a (PBP2a) [36, 37]. In our study, discrepancies between phenotypic oxacillin and cefoxitin susceptibility and the presence of *mec*A gene were observed for two and four isolates, respectively. The overall MRSA prevalence in this study was 30.6%. Oxacillin-resistant *S. aureus* are considered resistant to other β-lactam agents, i.e. penicillins, β-lactam/β-lactamase inhibitor combinations, cephems and carbapenems. This is because most cases of documented of MRSA infections have responded poorly to β-lactam therapy, or because convincing clinical data that document clinical efficacy for those agents have not been presented [20]. While all the MSSA strains tested in the present study were susceptible to cefoxitin and oxacillin, over half of them were found to be resistant to three antibiotics applied. Among the strains of MSSA, the most frequently observed resistance included lack of susceptibility to penicillin G, ampicillin and erythromycin. The present study confirmed the presence of the *mec*A gene responsible for resistance to methicillin in twenty-six of the *S. aureus* strains which were all resistant to penicillin G and ampicillin (Table 3). Resistance to methicillin is heterogeneous, which can cause some difficulty in determining these characteristics under in vitro conditions, and may lead to complications in the preparation of the test and the interpretation of results [19]. The literature data indicate that most multi-resistant strains of *Staphylococcus aureus* exhibit a lack of susceptibility to erythromycin and tetracycline, and less often to gentamycin and chloramphenicol [38]. Moreover, MRSA strains are also considered to be resistant to all cephalosporins and other β-lactam antibiotics regardless of the results of tests conducted under in vitro conditions [20]. In our study, the most frequently observed resistance among MRSA strains included lack of susceptibility to penicillin G, ampicillin, amoxicillin, cefoxitin, amoxicillin + clavulanic acid, oxacillin and tetracycline. Over half of the tested strains also showed a lack of sensitivity to erythromycin and clindamycin. The rates of resistance of MRSA to chloramphenicol, gentamycin and trimethoprim–sulphamethoxazole were much lower (< 30%) than those to other antibiotics.

These data are disturbing, given the fact that *S. aureus* strains were isolated from poultry flocks located only in the area of central-western Poland. For comparison, in research conducted by Persoons et al. (2009) in Belgium, MRSA was isolated from 8 broiler chickens from two of the 14 farms sampled. In the MRSA-positive flocks, the number of positive samples varied between 1/5 (20%) and 5/5 (100%) [4]. However, data published in 2009 by de Boer et al. indicate that the highest prevalence of MRSA in poultry was in the Netherlands, where MRSA was isolated from 16.0% of chicken meat samples and 35.3% of retail turkey meat samples [17]. Most MRSA isolates have been shown to be resistant to several classes of antibiotics and more than 80% of them produce penicillinases [19]. The research conducted on poultry farms indicate that the farm staff is exposed to an increased risk of MRSA colonization compared to the general population [39]. The results of the study published by Richter et al. indicate that the prevalence of MRSA in the investigated turkey meat production farms in the southwest of Germany reached 90%. In addition, among these isolates, the majority were livestock-associated MRSA [40].

Bacteriophages play an important role in the biology of *S. aureus*. Through horizontal gene transfer and lysogenic phage conversion associated with virulence factors, they can convert a non-virulent strain of staphylococcus into a virulent one [1, 19, 41]. Interestingly, the toxins of the superantigens are detected more often among methicillin-resistant staphylococci. For example, enterotoxin A is more commonly produced by MRSA strains [42]. The use of β-lactam antibiotics and fluoroquinolones in sub-inhibitory concentrations has been shown to induce the emergence of prophages from latent states. This results in the replication of the viral genome and the amplification of the genes encoded therein, including lysogenic conversion genes [12, 43].

The results of our study indicated a high prevalence of prophages among the test isolates of *S. aureus*. In all, 15 prophage patterns were observed among the isolates. The most prevalent prophage patterns or lysogenic types among MRSA isolates were single lysogenic 77-like, while among MSSA isolates the most prevalent prophage pattern was 11-like. The prophages detected least frequently were 3A-like (SGA) (5.8%) and 187-like (SGL) (3.8%) (Tables 4, 5). Similar results have been obtained by other authors, who studied the types of bacteriophages presented in human methicillin-resistant *S. aureus* strains [44]. However, the most prevalent prophage type detected in the genome of human MRSA strains by Pantůček et al. was 3A-like [31]. The difference may be due to the fact that the microorganisms were isolated in different geographic locations and from different animal species.

The results of our study indicate that as many as 18 (13 MRSA and 5 MSSA) of the strains tested had the *sak* gene responsible for the production of staphylokinase. Some authors believe that the activity of phage-encoded virulence factors such as staphylokinase is specific for human target molecules, indicating tight host/pathogen coevolution [45]. Matthews and Novick suggest that strains of bovine origin carry *sak*-containing phages less frequently than human isolates [46]. Our knowledge about prophage patterns in *S aureus* of poultry origin is poorly known. There have been reports suggesting that serogroup F (77-likevirus) and B (11-likevirus) phages of *Staphylococcus aureus* are capable of expressing staphylokinase [31, 47, 48,]. The results of our study indicate that most of the *S. aureus* strains that possessed the *sak* gene also had 77-like prophages incorporated into their genome.

As well as its importance as a livestock and community-associated pathogen, *S. aureus* is also a well-known cause of food intoxication [2, 3, 8, 49]. It is estimated that the actual number of foodborne illnesses caused by *S. aureus* is much higher than those reported [30]. The relationship between the production of enterotoxin A and the presence of bacteriophages and the enterotoxin A (*sea*) gene in the DNA of temperate bacteriophages was first described by Betley et al. [10]. Coleman et al. showed that, depending on the phenotype observed in lysogenic cells, bacteriophages converting enterotoxin A can simultaneously convert staphylokinase [16, 25]. Among the *S. aureus* strains tested, the presence of the *sea* gene was found in three strains (one MRSA and two MSSA), two of which also had the *sak* gene. Interestingly, strains with both the *sea* and *sak* genes had the same prophage pattern (3A-77a) (Tables 4, 5). A relationship between the production of other toxins (toxic shock syndrome and exfoliative toxins) and the presence of prophages in the genome of *S. aureus* bacteria has also been demonstrated [8]. In research conducted by El-Adawy et al. (2016), the genes encoding the toxic shock syndrome toxin (*tst*), *sea, seb, sec* and *see*, and genes for exfoliative toxins (*eta*/*etb)* were not found in any turkey and broiler chicken isolates [50]. In our study, the presence of genes (*tst-1*) was detected in three of the 85 strains tested, while only one strain had the gene responsible for producing exfoliatin A (*eta*). The MRSA strain that possessed the *tst-1* gene had the 3A-77a prophage pattern, while the other two MSSA strains had prophage patterns 11-like and 3A-11-77b. In contrast, the MSSA strain in which the exfoliatin A gene was detected had prophage pattern 11-77b (Tables 4, 5).

The results are particularly disturbing given that exfoliative toxins (ETs) produced by *S. aureus* strains are the major causative agents of blistering skin disorders in humans. Literature data indicate that staphylococcal scaled-skin syndrome (SSSS) caused by antibiotic-resistant strains of *S. aureus* has recently emerged as a serious problem [5].

The toxic shock syndrome toxin 1 (*tst-1)* gene is located within mobile genetic element such as staphylococcal SaP11 pathogenicity island, which by horizontal transfer can occur through bacteriophage transduction. Studies conducted on strains of *S. aureus* isolated from healthy humans showed that a significant percentage of isolates (24.3%) possessed the gene for TSST-1 [23]. Therefore, it is believed that many healthy individuals carry toxin-producing strains of *S. aureus*. Some pathogenicity islands of *S. aureus* can be transduced to other staphylococcal species: *S. chromogenes, S. intermedius, S. xylosus* and *S. epidermidis* [46]. The transfer of the SaPI1 pathogenicity island associated with prophage induction from the cells of *S. aureus* into *Listeria monocytogenes* has been observed as well, having spontaneously occurred in cow’s milk [11].

Pulsed-field gel electrophoresis (PFGE) is the most applied and effective genetic typing method for epidemiological studies and investigation of foodborne outbreaks caused by different pathogens, including *Staphylococcus aureus*. Traditionally, human MRSA isolates have been typed by pulsed-field gel electrophoresis (PFGE), using *Sma*I as the restriction enzyme [51]. The advantages of using PFGE are good discriminatory power and good reproducibility at the interlaboratory level when standardized protocols are used. It is possible that livestock-associated MRSA (LA-MRSA) is not typeable by this method, as the activity of *Sma*I is blocked due to methylation of the restriction site [52]. In our study, only three of the MRSA isolate was typeable by *Sma*I enzyme digestion. The remaining twenty-three isolates were typeable using the restriction enzyme *Apa*I, an alternative to *Sma*I as mentioned above. It is likely that the twenty-three MRSA isolates that have not been digested by the *Sma*I enzyme and have been digested with the *Apa*I enzyme are of animal origin. Similar observations were made by Bens et al. (2006) when examining their isolates collected at a pig farm [52].

DNA restriction analysis using *Apa*I as the restriction enzyme revealed sixteen different patterns, which means that in the case of MRSA there were six and three identical strains with the same macrorestriction profiles (Fig. 1). This may suggest a cross infection by these bacteria in different individuals within the poultry flock.

## Conclusions

We confirmed the presence of 30,6% positive strains of MRSA in food production animals (chickens and turkeys), which as livestock are in close contact with humans (farmers, farm co-workers, veterinarians). The results strongly suggest that people working with livestock are at a potential risk of becoming MRSA carriers and hence at an increased risk of infections caused by MRSA. This might complicate MRSA control measures in human healthcare, urging research into risk factors and transmission routes. Also, the relative high frequency of some virulence genes in strains of *S. aureus* originated from slaughtered poultry in this study may reflect the potential hazard to consumers.

The *S. aureus* strains we studied harboured at least one or up to three prophages. In consequence, high diversity among prophages results in the high potential of the isolate to produce a wide range of virulence factors. In our studies, the presence of 77-like prophages incorporated into bacterial genome was especially often demonstrated. Various authors emphasize the special role of these prophages in the spread of virulence factors (staphylokinase, enterotoxin A) between *Staphylococcus* strains [31, 48]. From the point of view of human medicine as well as veterinary medicine, it is also particularly disturbing that *S. aureus* virulence factors can be transferred via mobile genetic elements not only within strains of the same species but also between species and even types of bacteria.

## Electronic supplementary material

Below is the link to the electronic supplementary material.

Supplementary **Fig 1** Agarose gel electrophoresis showing multiplex PCR amplification products for the S. aureus enterotoxins genes. Lines M- DNA molecular size marker (Nova 100-bp DNA ladder - Novazym Polska); Lanes: 1, control strain FRI913; 2, control strain ATCC13566 ; 3, control strain FRI151m ; 4, seb; 5, sea; 6, sea; 7,*seb*; 8, sea; 9, sed; 10, sec; 11, seb; 12, sec. (TIF 3294 KB)

Supplementary **Fig 2** Agarose gel electrophoresis showing multiplex PCR amplification products for the prophage genes of *S. aureus* strains. Lines M- DNA molecular size marker (Nova 100-bp DNA ladder - Novazym Polska); Lanes: 1, control strain NCTC 8325; 2, double lysogenic 3A (SGA) - 11 (SGB); 3,double lysogenic 3A (SGA) - 11 (SGB); 4, double lysogenic 3A (SGA) - 11 (SGB); 5, double lysogenic 3A (SGA) - 11 (SGB); 6, double lysogenic 77a (SGFa) – 77b (SGFb); 7, double lysogenic 77a (SGFa) – 77b (SGFb); 8, double lysogenic 3A (SGA) - 11 (SGB); 9, triple lysogenic 3A (SGA) – 77a(SGFa)- 77b (SGFb); 10, triple lysogenic 3A (SGA) – 77a(SGFa)- 77b (SGFb); 11, triple lysogenic 3A (SGA)- 11 (SGB) – 77b (SGFb); 12, double lysogenic 77a (SGFa)- 77b (SGFb); 13, triple lysogenic 3A (SGA) – 77a(SGFa)- 77b (SGFb); 14, triple lysogenic 3A (SGA) – 77a(SGFa)- 77b (SGFb); 15, triple lysogenic 3A (SGA)- 11 (SGB) – 77b (SGFb); 16, quadruple lysogenic 3A-like (SGA) - 11-like (SGB) - 77a (SGFa) – 77b (SGFb) (TIF 3834 KB)

Supplementary **Fig 3** Agarose gel electrophoresis showing multiplex PCR amplification products for the *S. aureus* exfoliative toxins A and B (*eta, etb)*, and toxic shock syndrome toxin (*tst) genes*. Lines M- DNA molecular size marker (Nova 100-bp DNA ladder - Novazym Polska); Lanes: 1, control strain CCM7056; 2, control strain FRI913; 3, tst; 4, tst; 5, eta (TIF 1486 KB)

Supplementary **Fig 4** Result of pulsed field gel electrophoresis of the MRSA strains (reference: MRSA–43300 and MSSA–29213 and ours:(1-12) after digestion with the *Sma*I restriction enzyme. M–molecular weight standard (100 bp DNA ladder, MBI Fermentas, Lithuania) (TIF 5374 KB)

Supplementary **Fig 5** Result of pulsed field gel electrophoresis of the MRSA strains (13-26) after digestion with the *Sma*I restriction enzyme. M–molecular weight standard (100 bp DNA ladder, MBI Fermentas, Lithuania) (TIF 5196 KB)

Supplementary **Fig 6** Result of pulsed field gel electrophoresis of the MRSA strains (1-11) after digestion with the *Apa*I restriction enzyme. M–molecular weight standard (100 bp DNA ladder, MBI Fermentas, Lithuania) (TIF 5431 KB)

Supplementary **Fig 7** Result of pulsed field gel electrophoresis of the MRSA strains (12-20) after digestion with the *Apa*I restriction enzyme. M–molecular weight standard (100 bp DNA ladder, MBI Fermentas, Lithuania) (TIF 4440 KB)

Supplementary **Fig 8** Result of pulsed field gel electrophoresis of the MRSA strains (21-26) after digestion with the *Apa*I restriction enzyme. M–molecular weight standard (100 bp DNA ladder, MBI Fermentas, Lithuania) (TIF 2963 KB)
