# Supplementary figures and images for: Association Between the Methicillin Resistance of Staphylococcus aureus Isolated from Slaughter Poultry, Their Toxin Gene Profiles and Prophage Patterns
Source: Curr Microbiol. 2018 May 29;75(10):1256–66. doi: 10.1007/s00284-018-1518-9 (PMC6132865; doi:10.1007/s00284-018-1518-9)

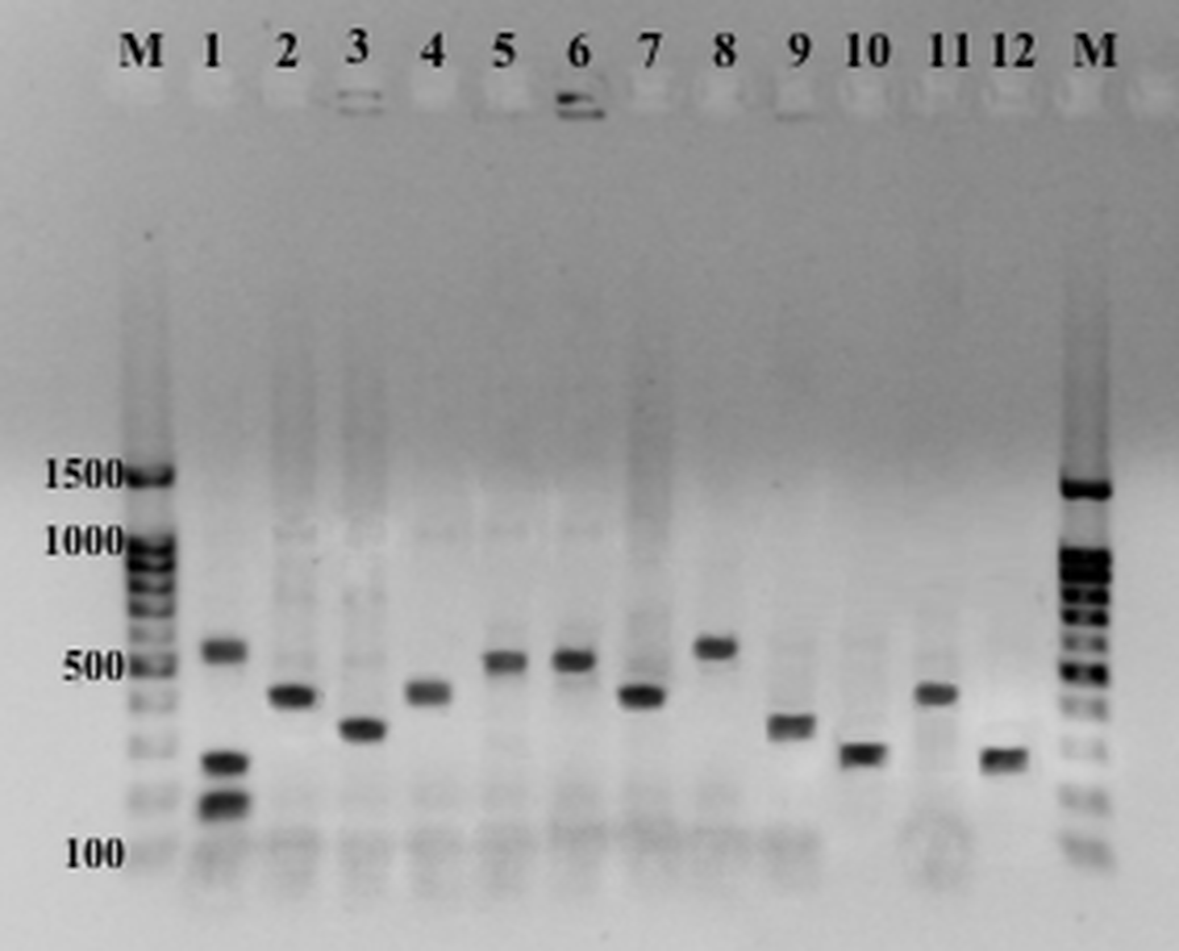

Supplement: Supplementary file 1 — Supplementary Fig 1 Agarose gel electrophoresis showing multiplex PCR amplification products for the S. aureus enterotoxins genes. Lines M- DNA molecular size marker (Nova 100-bp DNA ladder - Novazym Polska); Lanes: 1, control strain FRI913; 2, control strain ATCC13566 ; 3, control strain FRI151m ; 4, seb; 5, sea; 6, sea; 7,seb; 8, sea; 9, sed; 10, sec; 11, seb; 12, sec. (TIF 3294 KB) [file 284_2018_1518_MOESM1_ESM.tif]

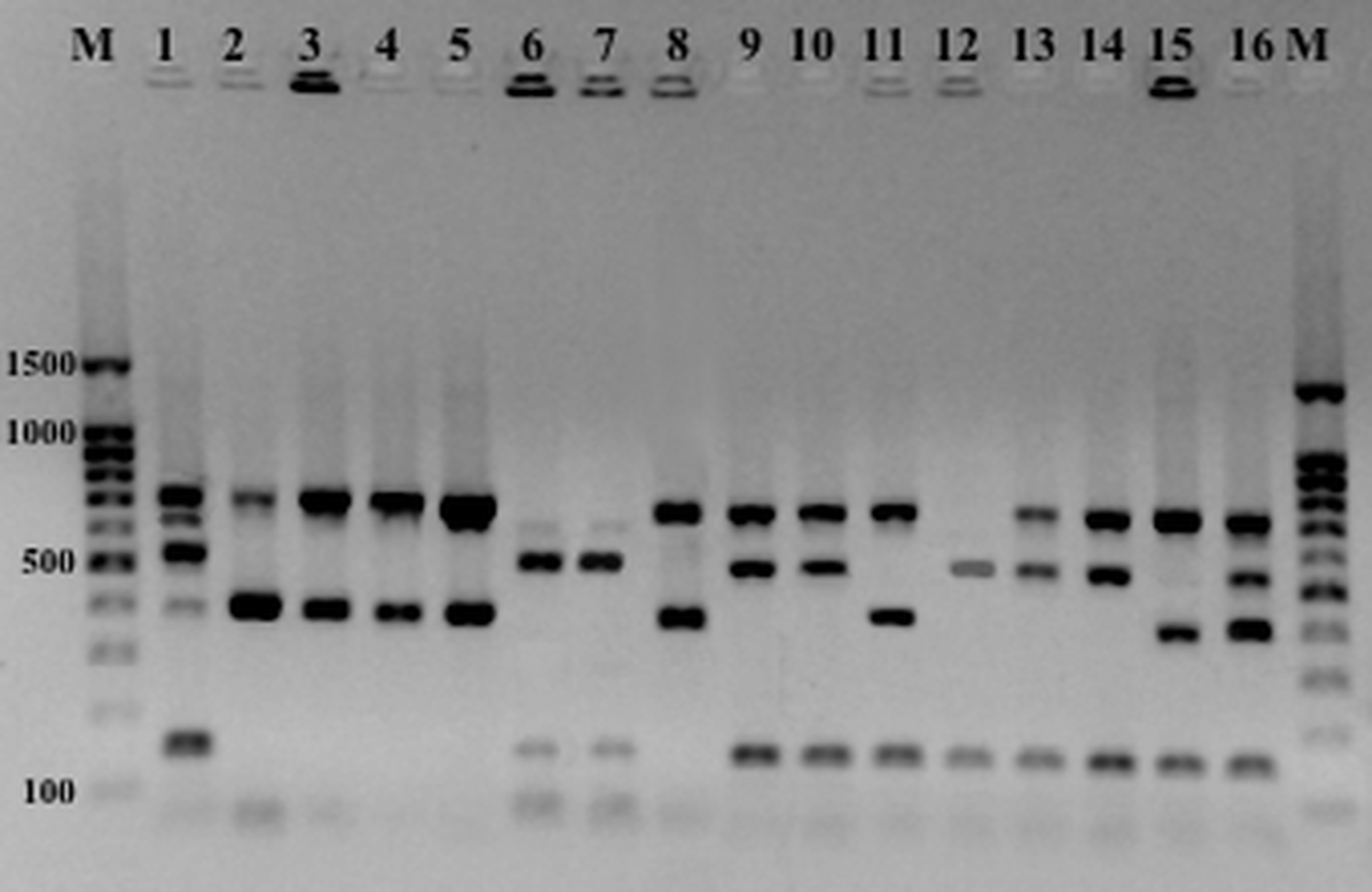

Supplement: Supplementary file 2 — Supplementary Fig 2 Agarose gel electrophoresis showing multiplex PCR amplification products for the prophage genes of S. aureus strains. Lines M- DNA molecular size marker (Nova 100-bp DNA ladder - Novazym Polska); Lanes: 1, control strain NCTC 8325; 2, double lysogenic 3A (SGA) - 11 (SGB); 3,double lysogenic 3A (SGA) - 11 (SGB); 4, double lysogenic 3A (SGA) - 11 (SGB); 5, double lysogenic 3A (SGA) - 11 (SGB); 6, double lysogenic 77a (SGFa) – 77b (SGFb); 7, double lysogenic 77a (SGFa) – 77b (SGFb); 8, double lysogenic 3A (SGA) - 11 (SGB); 9, triple lysogenic 3A (SGA) – 77a(SGFa)- 77b (SGFb); 10, triple lysogenic 3A (SGA) – 77a(SGFa)- 77b (SGFb); 11, triple lysogenic 3A (SGA)- 11 (SGB) – 77b (SGFb); 12, double lysogenic 77a (SGFa)- 77b (SGFb); 13, triple lysogenic 3A (SGA) – 77a(SGFa)- 77b (SGFb); 14, triple lysogenic 3A (SGA) – 77a(SGFa)- 77b (SGFb); 15, triple lysogenic 3A (SGA)- 11 (SGB) – 77b (SGFb); 16, quadruple lysogenic 3A-like (SGA) - 11-like (SGB) - 77a (SGFa) – 77b (SGFb) (TIF 3834 KB) [file 284_2018_1518_MOESM2_ESM.tif]

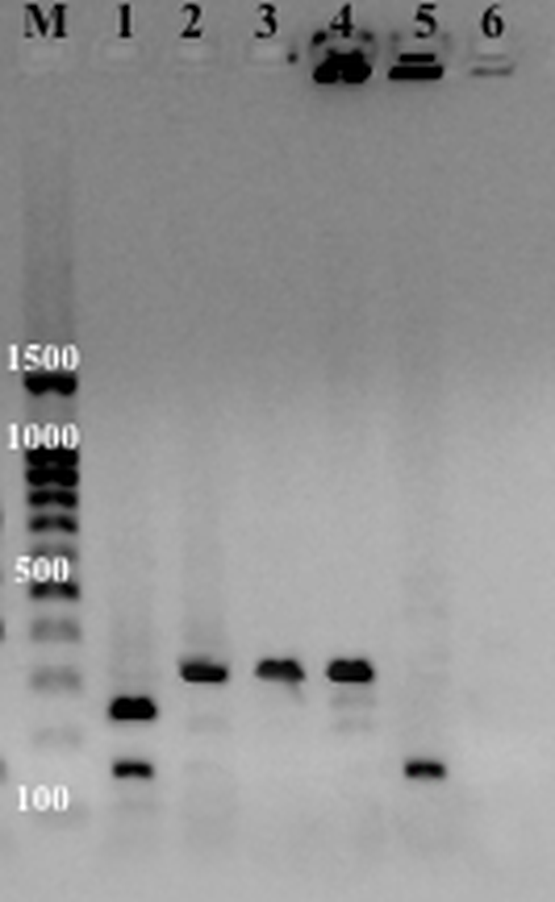

Supplement: Supplementary file 3 — Supplementary Fig 3 Agarose gel electrophoresis showing multiplex PCR amplification products for the S. aureus exfoliative toxins A and B (eta, etb), and toxic shock syndrome toxin (tst) genes. Lines M- DNA molecular size marker (Nova 100-bp DNA ladder - Novazym Polska); Lanes: 1, control strain CCM7056; 2, control strain FRI913; 3, tst; 4, tst; 5, eta (TIF 1486 KB) [file 284_2018_1518_MOESM3_ESM.tif]

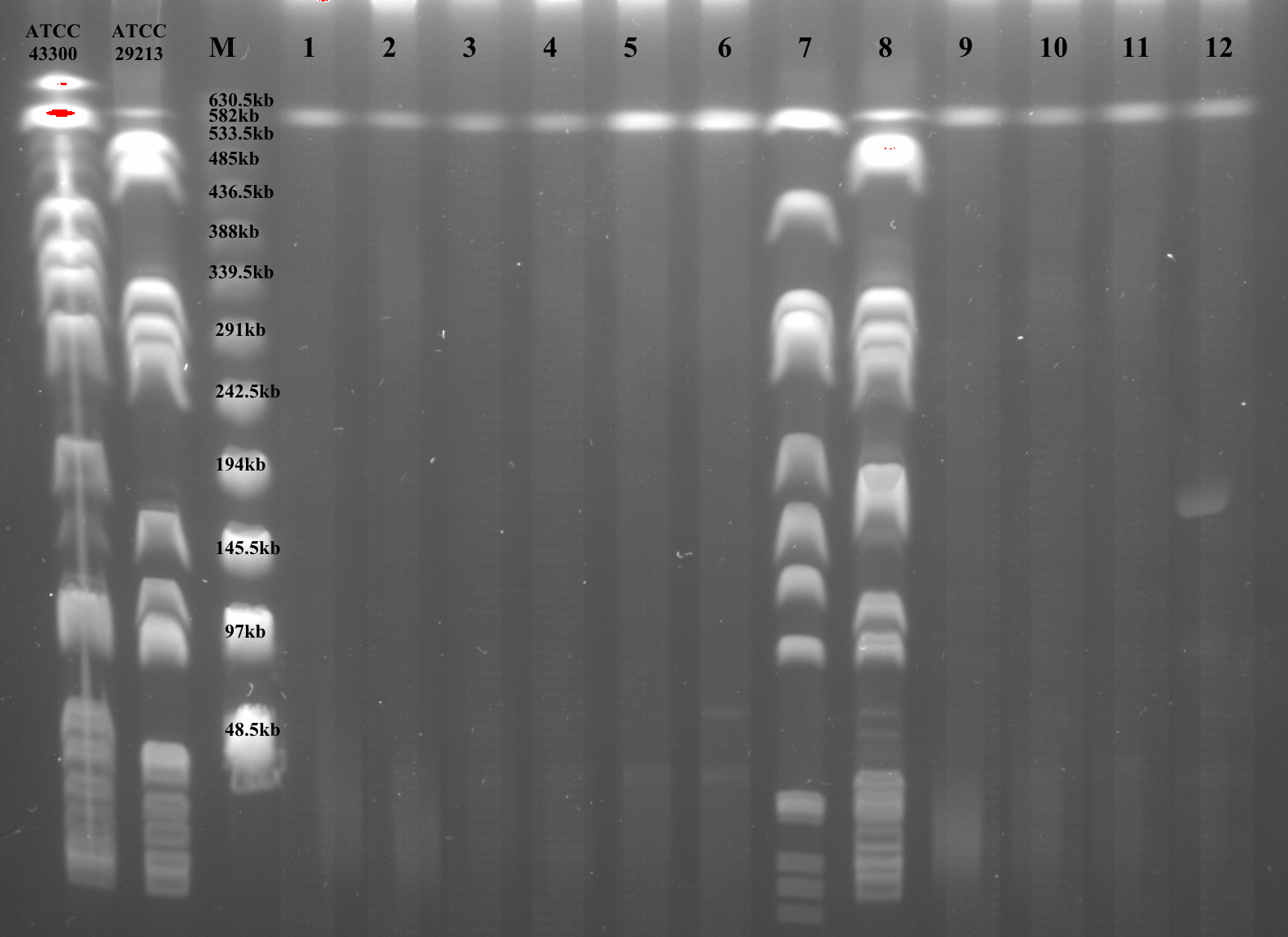

Supplement: Supplementary file 4 — Supplementary Fig 4 Result of pulsed field gel electrophoresis of the MRSA strains (reference: MRSA–43300 and MSSA–29213 and ours:(1-12) after digestion with the SmaI restriction enzyme. M–molecular weight standard (100 bp DNA ladder, MBI Fermentas, Lithuania) (TIF 5374 KB) [file 284_2018_1518_MOESM4_ESM.tif]

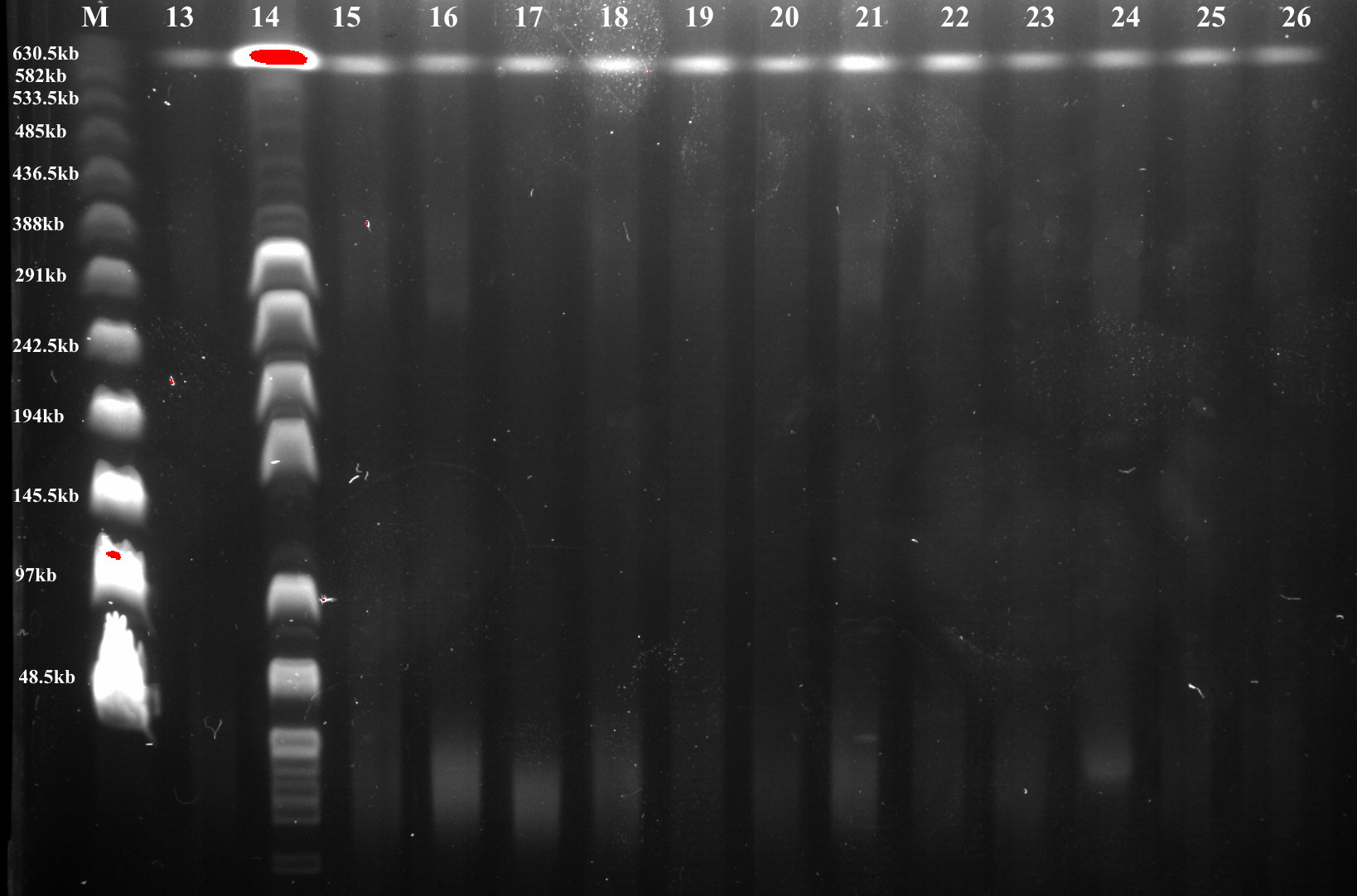

Supplement: Supplementary file 5 — Supplementary Fig 5 Result of pulsed field gel electrophoresis of the MRSA strains (13-26) after digestion with the SmaI restriction enzyme. M–molecular weight standard (100 bp DNA ladder, MBI Fermentas, Lithuania) (TIF 5196 KB) [file 284_2018_1518_MOESM5_ESM.tif]

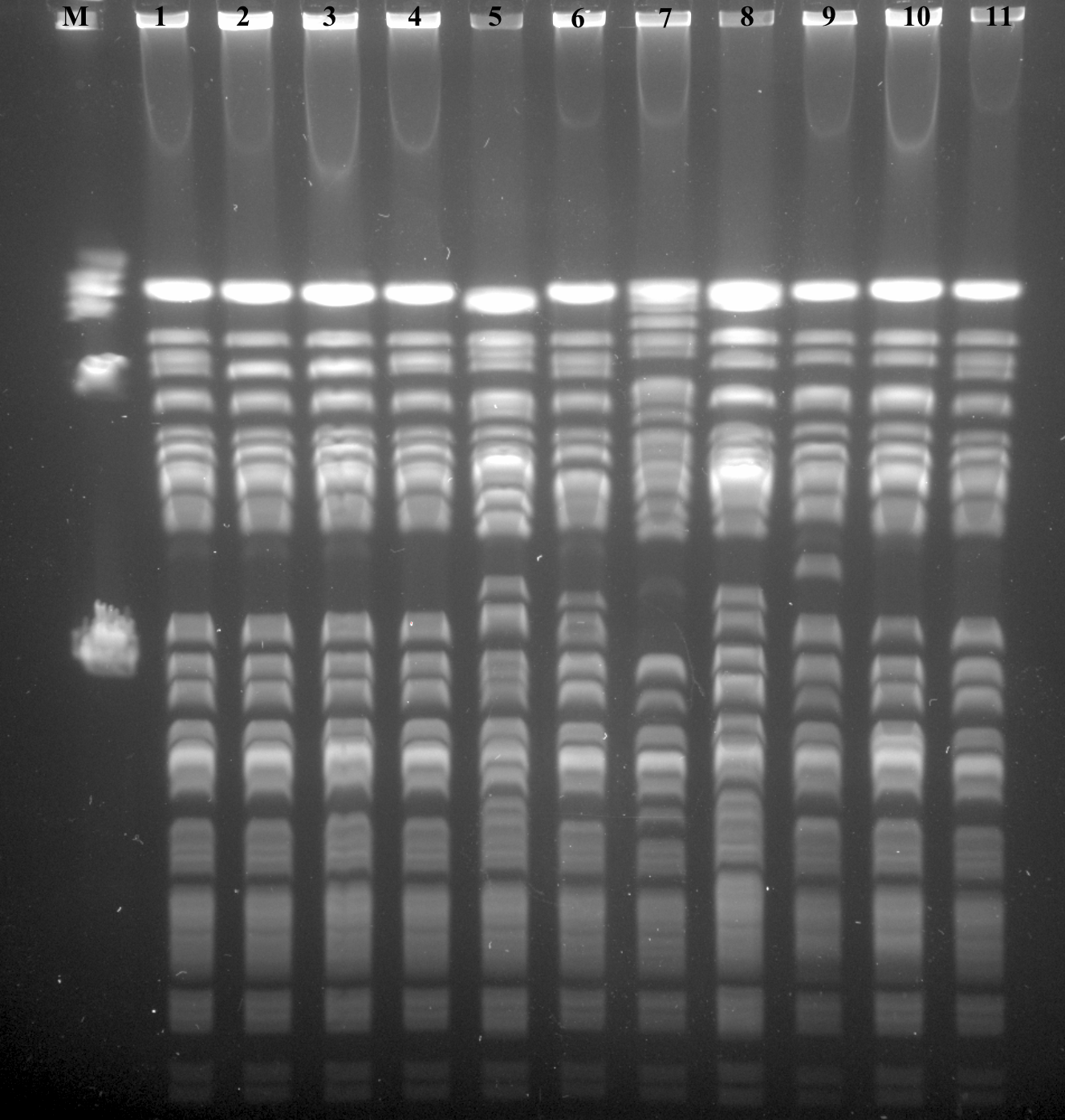

Supplement: Supplementary file 6 — Supplementary Fig 6 Result of pulsed field gel electrophoresis of the MRSA strains (1-11) after digestion with the ApaI restriction enzyme. M–molecular weight standard (100 bp DNA ladder, MBI Fermentas, Lithuania) (TIF 5431 KB) [file 284_2018_1518_MOESM6_ESM.tif]

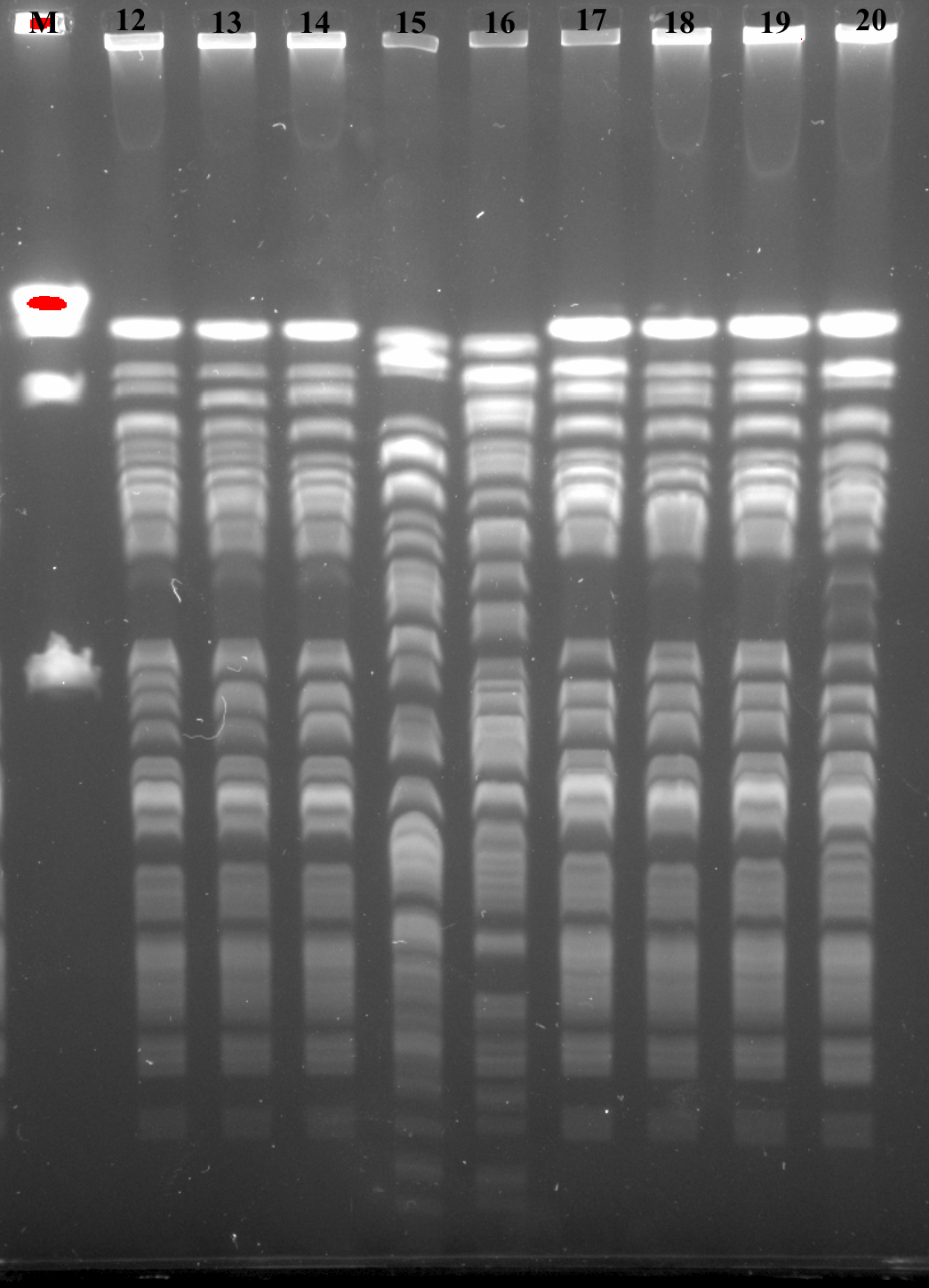

Supplement: Supplementary file 7 — Supplementary Fig 7 Result of pulsed field gel electrophoresis of the MRSA strains (12-20) after digestion with the ApaI restriction enzyme. M–molecular weight standard (100 bp DNA ladder, MBI Fermentas, Lithuania) (TIF 4440 KB) [file 284_2018_1518_MOESM7_ESM.tif]

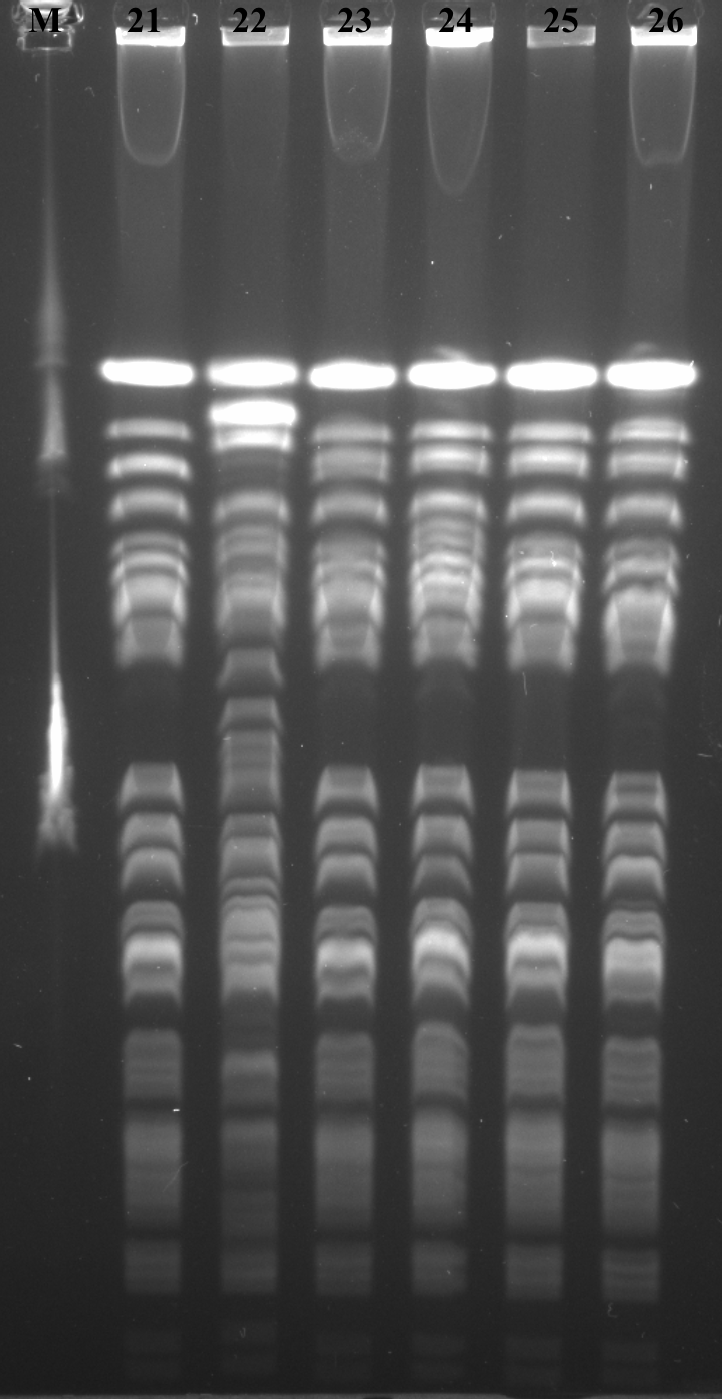

Supplement: Supplementary file 8 — Supplementary Fig 8 Result of pulsed field gel electrophoresis of the MRSA strains (21-26) after digestion with the ApaI restriction enzyme. M–molecular weight standard (100 bp DNA ladder, MBI Fermentas, Lithuania) (TIF 2963 KB) [file 284_2018_1518_MOESM8_ESM.tif]
